# Bioremediation of Neonicotinoid Pesticide, Imidacloprid, Mediated by *Bacillus cereus*

**DOI:** 10.3390/bioengineering10080951

**Published:** 2023-08-10

**Authors:** Farah Naz Talpur, Ahsanullah Unar, Sana Kanwal Bhatti, Laila Alsawalha, Dalia Fouad, Humaira Bashir, Hassan Imran Afridi, Farid Shokry Ataya, Ohoud A. Jefri, Muhammad Sohail Bashir

**Affiliations:** 1 National Centre of Excellence in Analytical Chemistry, University of Sindh, Jamshoro 76080, Pakistan; sanasanakanwal3@gmail.com (S.K.B.); hassanimranafridi@yahoo.com (H.I.A.); 2 School of Life Sciences, University of Sciences and Technology of China, Hefei 230052, China; aunar@mail.ustc.edu.cn; 3 Faculty of Science, Zarqa University, Zarqa 13110, Jordan; lsawalha@zu.edu.jo; 4 Department of Zoology, College of Science, King Saud University, P.O. Box 22452, Riyadh 11495, Saudi Arabia; dibrahim@ksu.edu.sa; 5 Department of Botany, University of the Punjab, Quaid-e-Azam Campus, 54590 Lahore, Pakistan; humaira_bashir96@yahoo.com; 6 Department of Biochemistry, College of Science, King Saud University, P.O. Box 2455, Riyadh 11451, Saudi Arabia; fataya@ksu.edu.sa; 7 Department of Biological Science, Faculty of Science, King Abdul-Aziz University, Jeddah 2158, Saudi Arabia; 8 Institutes of Physical Science and Information Technology, Key Laboratory of Structure and Functional Regulation of Hybrid Materials of Ministry of Education, Anhui University, Hefei 230601, China; 9 Department of Polymer Science and Engineering, University of Science and Technology of China, Hefei 230026, China

**Keywords:** imidacloprid, *Bacillus cereus*, degradation, metabolites

## Abstract

Imidacloprid, a toxic pesticide of the chloronicotinyl category, is employed extensively in agricultural fields, and its exposure causes serious health issues. Biodegradation is considered to be a green and economical approach to remediate pesticides. Herein, imidacloprid degradation efficiency of *Bacillus* sp. is highlighted, among which *Bacillus cereus* exhibited the greatest degradation; optimization of experimental variables (pH, imidacloprid and agitation time) via Box–Behnken factorial design and analysis of variance (ANOVA) revealed 92% biodegradation at the initial substrate concentration of 0.03 mM, aerobically in 11 days under favorable pH 7. The subsequent metabolites, identified through liquid chromatography–mass spectrometry, were 5-hydroxy imidacloprid, imidacloprid-guanidine and 6-chloronicotinic acid.

## 1. Introduction

Pesticides are intensively used in our environment, and more than 500 pesticides have been reported, particularly in the field of agriculture. Annually, around two million tons of pesticides are utilized worldwide [1], and unfortunately, only 0.1% reach their targets, while the rest of them migrate to soil, air and water resources [2], among which the greatest concern regarding human exposure to pesticides is their presence in water [3,4,5].

Imidacloprid [1-(6-chloro-3-pyridylmethyl)-N-nitroimidazolidin-2-ylideneamine] is the first member of chloronicotinyl insecticides and considered to be a comparatively new class of pesticides [6]. Since its introduction in 1991, products containing imidacloprid have gained registration in about 120 countries and marketed for more than 140 agricultural crops [7]. Imidacloprid was the most commonly used insecticide in the world from 1999 until at least 2018 [8]. The pesticide has a partial approval in the U.S. and other countries where it is widely used, despite being banned for all outdoor usage in the entire European Union since 2018 [9]. Pakistan is the second-highest consumer of pesticides among the South Asian nations, and the agriculture sector is where these pesticides are most frequently employed [10,11]. Among pesticides, imidacloprid is the fourth-ranking insecticide (7.6% by weight) in the country [12] and is employed to control termites, sucking insects, soil insects and some species of chewing insects [13].

Its characteristics like fair solubility, low volatility [14] and high leaching ability make it a potential contaminant of surface and underground water [3,15], as also documented by the Environmental Protection Agency (EPA) [16]. The World Health Organization and the United States Environmental Protection Agency have rated imidacloprid as “moderately toxic” on an acute oral basis to mammals and low-toxicity on a dermal basis [17]. Imidacloprid is a highly relentless pesticide with a hydrolysis half-life of 33–44 days and a photolysis half-life of 39 days at the soil surface, while in soil, its half-life is 26.5–229 days [18]. According to an assessment of 1900 emergency cases in a hospital in Pakistan, 40% of them were due to acute poisoning, with organophosphates being responsible for both the greatest number of poisoning cases and the highest mortality rates [19]. In an ICU study conducted in Karachi, organophosphate poisoning patients had a mortality rate of 7.69%, with acute respiratory distress syndrome secondary to aspiration pneumonia listed as the cause of death [20].

Imidacloprid residues have been detected in vegetables, soil and water in southern Punjab, Pakistan [11]. In addition, Millot et al., in 2014 reviewed the 103 wildlife mortality incidents reported by the French SAGIR Network from 1995 to 2014, for which toxicological analyses detected imidacloprid residues [21].

Imidacloprid is considered among the top six highly dangerous pesticides [22], and its exposure causes labored breathing, twitching, thyroid lesions, affected reproduction, reduced weight, cardiovascular and central nervous system disorders [16,23]. It is also toxic to some beneficial organisms like birds, bees, earthworms, shrimps, crustaceans and fish, etc. [24].

To remove pesticides, biodegradation is a green and economical approach using biological mediators [25], different microbes including *Bacillus* species have been reported for pesticide degradation as [26] published the potential of *Bacillus thuringiensis* NCIM 2159 and *Proteus* spp. SUK 7 for degradation (7.59–100%) of 11 different pesticide residues, including imidacloprid, in lake water. Another *Bacillus* species, *B. pumilus* strain BFB30, assimilated carbendazine up to 73.4% [15]. Similarly, Zhao and Wang [27] reported *Lactobacillus bulgaricus* for the degradation of dimethoate, fenthion and monocrotophos and *L. plantarum* for malathion, methyl parathion and trichlorphon degradation in skimmed milk. *Bacillus* species will be investigated in the current study owing to their ability to produce a wide range of enzymes and secondary metabolites that can degrade or modify pesticide molecules. Additionally, Bacillus species demonstrate high tolerance to elevated pesticide concentrations and can survive in diverse environmental conditions [28]. The optimization of the biodegradation process involves the investigation of experimental variables such as pH, concentration, time and other factors related to studies. Factorial designs are preferred over classical optimization methods due to their comprehensive, efficient and robust approach to experimental exploration and optimization [29]. To achieve this objective, strategies like full factorial, Box–Behnken and central composite designs are frequently employed [30]. Hence, keeping in view the toxicity and persistence of imidacloprid and the prevalence of biodegradation capability of *Bacillus species*, the present study was designed to assess the efficiency of different *Bacilli* for imidacloprid degradation, optimization of experimental variables (pH, agitation time and concentration) by Box–Behnken design and identification of degradation metabolites.

## 2. Materials and Methods

Imidacloprid (99.9% pure) was purchased from Sigma-Aldrich (St. Louis, MO, USA). Sodium hydroxide, hydrochloric acid, De Man Rogosa Sharpe (MRS) broth, mineral salt medium (MSM) and minimal salt medium were obtained from Oxoid Ltd. (Basingstoke, Hampshire, ENG). HPLC-grade acetonitrile from Scharlau Chemie S.A (Sentmenat, Spain) and analytical-grade ethyl acetate and isopropanol were bought from Mikrochem (Pezinok, Slovakia).

### 2.1. Microbial Strains

The screening panel consisted of four different *bacillus* strains *Lactobacillus plantarum* (GenBank accession no. KC288535), *Bacillus cereus* (GenBank accession no. DQ339674), *Bacillus thuringiensis* (from chili field soil; GenBank accession no. JQ579628) and *B. thuringiensis* (from cotton field soil; GenBank accession no. KF218168) isolated previously in our lab [31,32,33].

### 2.2. Preparation of Growth Culture

For biodegradation experiments, liquid cultures of *B. cereus* and *B. thuringiensis* were prepared in 100 mL mineral salt media (MSM) containing (L^−1^) K_2_HPO_4_ 0.2 g, MgSO_4_·7H_2_O 0.5 g, (NH_4_)_2_SO_4_ 1 g, KH_2_PO_4_ 0.8 g, FeSO_4_ 0.01 g, CaCl_2_ 0.05 g, Na_2_B_4_O_7_·10H_2_O 0.021 g, ZnCl_2_ 0.023 g, NiSO_4_ 0.032 g, (NH_4_)_6_Mo_7_O_24_·H_2_O 0.0144 g, CoCl_2_·H_2_O 0.021 g, MnCl_2_·4H_2_O 0.03 g and CuCl_2_·2H_2_O 0.01 g in sterilized water at pH 7.0, maintained by NaOH (0.1 M) and HCl (0.1 M). The autoclaved (121 °C) media was inoculated from fresh agar Petri dishes of corresponding microbes under aseptic conditions followed by 24 h agitation (150 rpm) at 30 °C. Liquid culture (100 mL) of *L. plantarum* was prepared in sterilized MRS (5 g) aqueous solution under identical conditions. The MRS was composed of essential nutrients, such as peptone (1.0%), meat extract (0.8%), yeast extract (0.4%), glucose (2.0%), K_2_HPO_4_ (0.2%), NaCH_3_COO·3H_2_O (0.5%), C_6_H_17_N_3_O_7_ (0.2%), MgSO_4_·7H_2_O (0.02%) and MnSO_4_·4H_2_O (0.005%).

### 2.3. Screening of Microbes

Before experimental use, the bacterial strains were aerobically pre-cultured overnight in 30 mL MRS broth and MSM by inoculation (6.25% *v/v*) from activated culture and supplemented with imidacloprid (0.0216 mM). Blank assays were also performed simultaneously in a similar manner without inoculation. All reactions were agitated (150 rpm) in a mechanical shaker at 30 °C for 7 days. The reaction mixtures were extracted with ethyl acetate and subjected to analyses.

### 2.4. Optimization of Experimental Variables

To find the favorable reaction conditions, the influence of various parameters was investigated. Since pH has a broad impact upon the whole-cell catalytic reactions [34], therefore first the pH influence was ascertained in the range of 5 to 9. The impact of substrate concentration (0.01 to 0.08 mM) was analyzed, keeping other reaction conditions constant, while the optimal biodegradation time for 0.03 mM imidacloprid was determined in the range of 1 to 25 days at pH 8. All experiments were carried out in triplicate, and error bars in figures show the standard error of the mean values.

### 2.5. Conditions for HPLC Analysis

**Figure 1:**
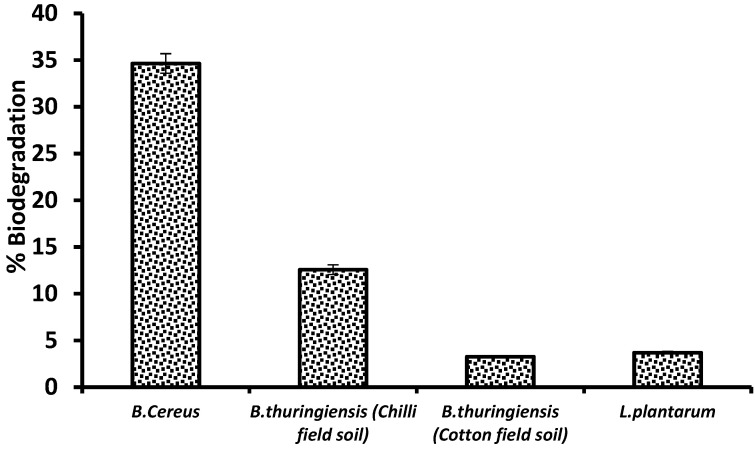
Screening of *Bacillus strains* for degradation potential of imidacloprid.

The biodegradation was monitored using a high-performance liquid chromatograph (HITACHI, UV 6000LP series) equipped with UV-detector. The analytes in a 20 µL injected sample were separated with a C18 column (250 × 4.6 mm × 5 µm) using a mixture of acetonitrile and water (60:40, *v/v*) with a flow rate of 1 mL min^−1^. The detection wavelength was set to 270 nm. The decrease in the imidacloprid peak area indicated its metabolization. (Figure 1).

### 2.6. LC-MS/MS Conditions

To recognize the imidacloprid degradation products, high-resolution electrospray mass spectrometry was performed with Agilent (Model 6460) triple quadruple liquid chromatography/mass spectrometer (LC-MS/MS). The experiments were conducted in the ESI interface with nebulizer gas (45 psi, 12 mL min^−1^). The capillary voltage was kept at 3500 V, and the MS/MS scan was completed in the range of 50 to 500 m/z.

### 2.7. Experimental Design/Statistical Analysis

The concept of design of experiments (DOE) involves a structured strategy for investigating the connection between different factors that impact the outcome of a particular process. Many researchers favor the Box–Behnken design (BBD) due to its simplicity, involving a limited number of experimental iterations for fine-tuning process parameters [35,36]. In this study, the BBD approach was employed to execute the effect of experimental variables such as pH (A), imidacloprid concentration (B) and agitation time (C) on the response variables, i.e., biodegradation of imidacloprid. Each experimental variable has three levels, −1, 0 and 1. Utilizing the values encoded as per Table 1, a total of 13 experiments were conducted to systematically refine the process variables, as outlined in Table 2.

The connection between the responses corresponds to the coded variables (xi, where i = 1, 2, 3 and so forth) established through the second-degree polynomial Equation (1).

Y = b_0_ + b_1_x_1_ + b_2_x_2_ + b_3_x_3_ + b_12_x_1_x_2_ + b_13_x_1_x_3_ + b_23_x_2_x_3_ + b_11_*x*^2^ + b_22_x^2^ +b_33_x^2^ (1)

As per the analysis of variance (ANOVA), the importance of the factors was evaluated, and their interaction was examined. The effectiveness of the polynomial model equations was gauged by the significance of the model in terms of calculating the degree of freedom, sum of squares, coefficient of determination R^2^, the adjusted coefficient of determination adj. R^2^ and F test [30]. Only the terms that exhibited significant impacts at a probability level of *p* < 0.05 were retained. The significant variables were subjected to numerical optimization, which was carried out at least three times to identify the best possible configuration. The most favorable conditions were determined using regression analysis in conjunction with three-dimensional response surface plots.

## 3. Results and Discussion

### 3.1. Screening of Bacillus Strains

**Figure 2:**
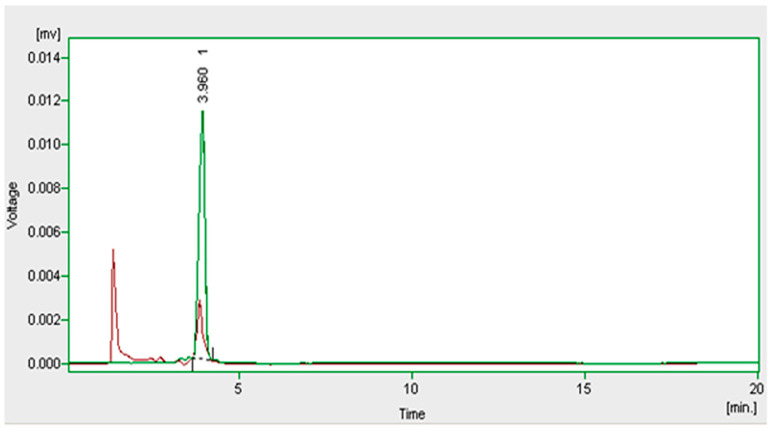
Overlay of HPLC chromatograms of imidacloprid biodegradation with *B. Cereus*, control (green) and reaction (red).

Four different bacillus strains, *B. cereus*, *B. thuringiensis* (from chili field soil) *B. thuringiensis* (from cotton field soil) and *L. plantarum*, were assessed for the degradation of imidacloprid, among which the most significant biocatalyst (*p* < 0.05) was found to be *B. cereus* with 34.64% degradation (Figure 1) and selected for the optimization of experimental variables. The decrease in the imidacloprid peak area indicated its metabolization (Figure 2).

Asim et al. [37] characterized the novel pesticide-degrading bacterial strains from industrial waste. A total of approximately 20 different strains were isolated, out of which six demonstrated significant pesticide biodegradation activity. Through 16S rRNA analysis, two of the isolated bacteria were identified as *Acinetobacter baumannii* (5B) and *Acidothiobacillus ferroxidans*, while the remaining four were identified as various strains of *Pseudomonas aeruginosa*. Among the *Pseudomonas aeruginosa* strains, 1A and 4D exhibited the highest degradation percentage of approximately 80% for DDT, while strain 3C showed the highest degradation percentage of 78% for aldrin. As for malathion, *A. baumannii* and *A. ferroxidans* demonstrated considerable degradation percentages of 53% and 54%, respectively. However, it should be noted that the degradation rate can vary depending on both the type of bacteria and the composition of the pesticide, indicating the need for further exploration.

### 3.2. Optimization of Experimental Variable with Box–Behnken Design

Table 2 represents the 13 experiments that were carried out in accordance with the design. The effect of input factors including temperature (A), pH (B), imidacloprid concentration (C) and agitation time on response compressive strength was evaluated. Box–Behnken designs stand as highly effective response surface designs, offering insights solely into the influence of experimental variables and overall experimental inconsistency using a minimal set of necessary trials. These designs exhibit excellent balance and rotational properties, while also demanding fewer experimental runs compared to widely used central composite designs, thereby yielding maximal information [38].

The predictive model in terms of actual factors for imidacloprid degradation is indicated in Equation (2).

Imidacloprid Biodegradation (%) = +92 + 7.375A − 7.5B + 26.875C − 2.5AB + 0.25AC + 6BC − 25.125A² − 9.375B² + 21.125C²(2)

The regression coefficients for biodegradation (%) are shown in Table 3. In this case, A, B, C, A^2^ and C^2^ are significant model terms. Values greater than 0.05 indicate the model terms are not significant. The model F-value of 29.92 implies the model is significant. There is only a 0.88% chance that an F-value this large could occur due to noise. A positive sign in coefficient of variable factors denotes a synergistic effect, whereas a negative sign denotes a factor’s antagonistic impact on the chosen response. The R^2^ value of 0.989 agreed with the adjusted R^2^ of 0.956. Adequate precision measures the signal-to-noise ratio. A ratio greater than four is desirable. A ratio of 16.15 indicates an adequate signal.

**Figure 3:**
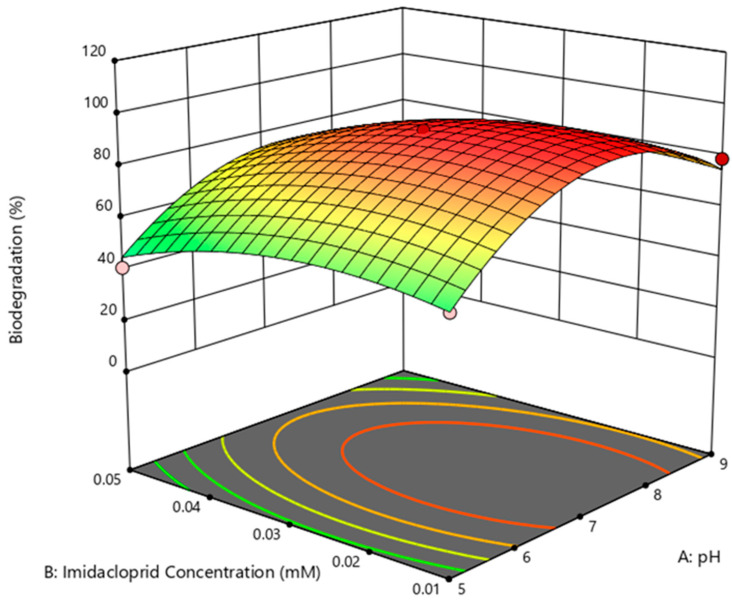
Response surface plot for biodegradation (%) as a function of pH and imidacloprid concentration mediated by *B. cereus*.

This model can be used to navigate the design space. The 3D-surface image (Figure 3) showed that pH and imidacloprid concentration changes had a greater effect on biodegradation.

It is beyond doubt that the biocatalysts are pH-specific in their functions and work at neutral or near-neutral pH [39]. Figure 3 depicts that the maximum degradation was achieved at pH 7, above which it declined abruptly (*p* < 0.05). The obtained optimal pH was in agreement with the literature as the degradation of imidacloprid [40] and acetamiprid [41] has been described at respective pHs of 7.9 and 7.7 using bacterial strains of *B. aerophilus*, *B. alkalinitrilicus* and *Rhodococcus* sp.

Li et al. [42] conducted a study to examine the impact of pH levels ranging from 5.0 to 8.0 on the degradation of omethoate, which is an acute organophosphorus pesticide. The most effective degradation was observed at pH 7.0, where the degradation rate increased rapidly over time, reaching 77.11% within 5 days. Both acidic and alkaline conditions were found to inhibit the growth of *Bacillus* sp. *YB-10*. When the pH was set to 5.0 during the degradation process, the degradation rates of omethoate were less than 40%.

**Figure 4:**
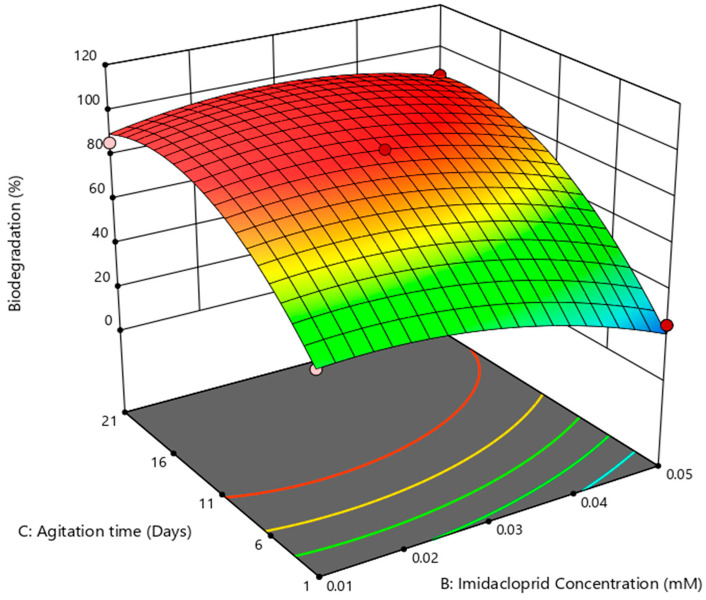
Response surface plot for biodegradation (%) as a function of imidacloprid concentration and agitation mediated by *B. cereus*.

The effect of substrate concentration was studied in the range of 0.01 to 0.05 mM. It is obvious from Figure 4 that a maximal degradation of 92% was observed at 0.03 mM in 11 days. Above that concentration, the degradation declined drastically (*p* < 0.05), the plausible reason behind which may be the toxic effects of imidacloprid as [43] reported with an increase in dose and durational exposure to imidacloprid, which decreases the growth of *Brevundimonas* sp. MJ15 by inhibiting nucleic acid and protein production.

Guo et al. [44] assessed the ability of the oligotrophic bacterial strain *Hymenobacter latericoloratus* CGMCC 16346 to break down imidacloprid through a process called co-metabolism hydroxylation. The bacterial cells were able to degrade 64.4% of imidacloprid at a concentration of 100 mg/L in surface water. In a separate study, neonicotinoids such as acetamiprid, imidacloprid and thiamethoxam, along with other emerging pollutants of both organic and inorganic nature, were successfully eliminated using a monoculture of *Chlorella vulgaris* and a mixed culture of microalgae and bacteria for traditional wastewater treatment. The findings of this study also indicated that even at environmentally relevant concentrations ranging from 1 to 20 µg/L, the growth of both microalgae and *C. vulgaris* cultures was not inhibited [45].

**Figure 5:**
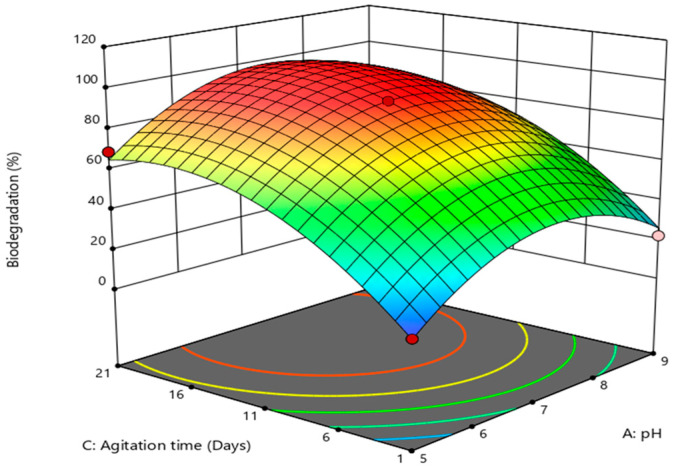
Response surface plot for biodegradation (%) as a function of agitation time and pH mediated by *B. cereus*.

The influence of agitation time and pH is shown in Figure 5, as is clear from the results showing that agitation time has a detrimental effect on biodegradation of imidacloprid. Optimal biodegradation was achieved in 11 days at pH 7; further increase in time led to a significant decrease (*p* < 0.05) in degradation, behind which the toxic behavior of the substrate can be envisaged because most of the aromatic compounds are toxic to the microorganisms [46,47,48,49]. Here, it is chiefly notable that in 1 day also, a remarkable degradation of 48% was accomplished at pH 7 with 0.01 mM imidacloprid.

Here, it can be reasoned that *B. cereus* has a high degradation efficiency; comparison of our results with the literature reflects that the achieved biodegradation was superior to that reported in the literature like consortium of *Achromobacter* sp. strain R-2079, Pseudomonas sp. HY8N and *Microbacterium* sp. B-2013 [15] accomplished 82% imidacloprid degradation in a comparatively long time period (20 days). Similarly, Sharma et al., [40] reported 5.83 mg/kg of the applied imidacloprid (100 mg/kg) degradation by consortium of *Bacillus aerophilus* and *Bacillus alkalinitrilicus* in 56 days. They [50] achieved almost complete degradation (97.47%) of imidacloprid (150 mg/kg) also by *B. aerophilus* in same reaction interval. In another study [51], just 43% imidacloprid degradation was accomplished by two soil-free enrichment cultures in 18 days.

There are, obviously, some limitations of using biological methods as they are time-consuming and less efficient compared to physicochemical methods [52]. Technologies based on biodegradation of organic pollutants are mostly intended for decontaminating small amounts of chemical substances spread in soil or water [53].

### 3.3. Identification of Imidacloprid Metabolites

**Figure 6:**
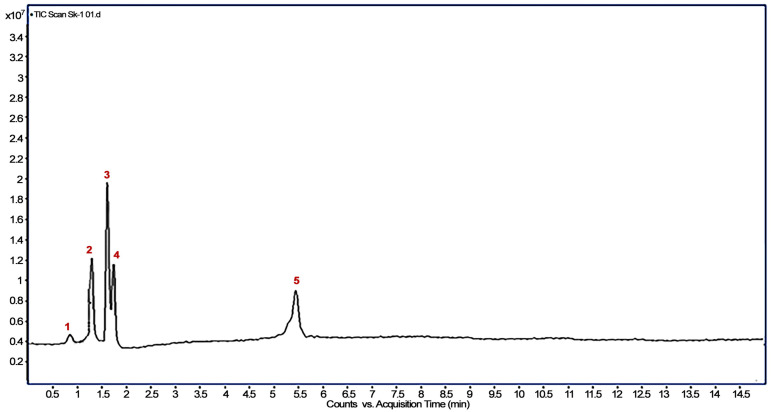
Total ion chromatogram of imidacloprid metabolites. Peak 1 represents solvent, peak 2 is of imidacloprid guanidine, peak 3 corresponds to 5−hydroxy imidacloprid and peak 4 is of 6−CNA, while peak 5 shows imidacloprid.

**Figure 7:**
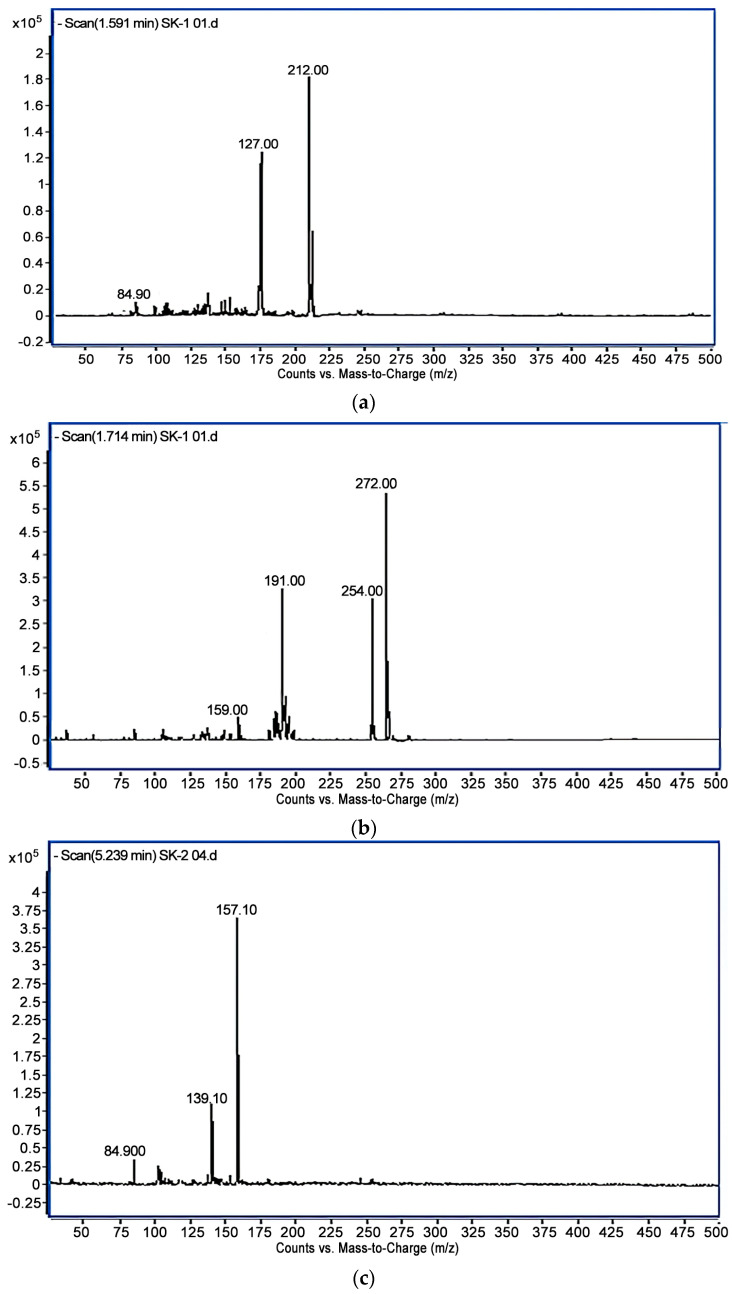
(**a**) Mass spectrum of imidacloprid−guanidine, where the molecular ion peak at 212 m/z is of imidacloprid-guanidine, while the peak at 127 m/z was formed due to removal of imidazolidinimine (C_3_H_7_N_3_) moiety. (**b**) Mass spectrum of 5−hydroxy imidacloprid, where the molecular ion peak at 272 corresponds to 5-hydroxy imidacloprid, and the fragment ion peaks at 254 and 191 m/z exhibit the loss of hydroxyl and N_2_O ions. (**c**) Mass spectrum of 6−CNA, where the molecular ion and fragment ion peaks at 157, 139 and 85 m/z represent the formation and cleavage of 6−CNA.

Three metabolites were identified based on their m/z and relative abundance (Figure 6 and Figure 7), viz., imidacloprid guanidine, 5−hydroxy imidacloprid and 6−chloronicotinic acid (6−CNA).

**Figure 8:**
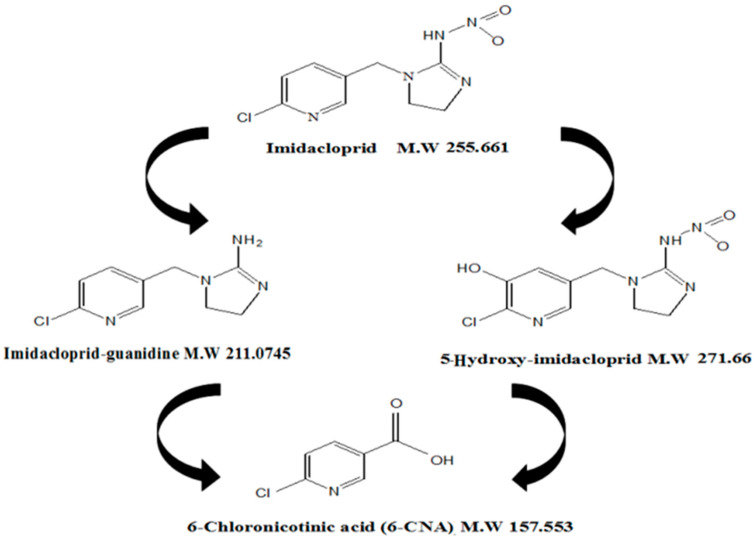
Structures and molecular weight of imidacloprid metabolites detected by LC-MS-MS analysis.

The LC-MS/MS results revealed that imidacloprid was transformed to 6−CNA via guanidine and 5−hydroxy imidacloprid intermediates (Figure 8). These findings are in agreement with previous studies, which proposed possible microbial metabolites, including imidacloprid−guanidine, imidacloprid−guanidine olefin and imidacloprid−urea [54]. The biodegradation of imidacloprid by a consortium of two Bacillus species was followed by the formation of 6−CNA and imidacloprid-nitrosoguanidine metabolites [40], while the formation of olefin and 5-hydroxy imidacloprid was also detected during the course of biodegradation [55,56]. *Pseudoxanthomonas indica*, imidacloprid−degrading bacteria, was identified by the authors from soil. Nuclear magnetic resonance analysis and liquid chromatography–mass spectrometry were used to identify two metabolites as olefin and 5−hydroxy imidacloprid, respectively [50]. In a lab setting, a study of imidacloprid biodegradation with *Bacillus aerophilus* in soil was conducted [51]. Urea and olefin had the highest metabolite values, while all treatments with amended soil also showed significant levels of 5−hydroxy, 6−chloronicotinic acid (6−CNA), nitrosimine and nitroguanidine (NTG).

## 4. Conclusions

Soil dwelling bacteria of the genus *bacillus* are capable of converting pesticides into simpler residues. In the present investigation, *B. cereus* was also found to be a versatile catalyst for the degradation of imidacloprid. The microbe was capable of metabolizing 92% imidacloprid in 11 days’ reaction time at neutral pH via efficient optimization with the Box–Behnken design through the formation of three metabolites which transformed imidacloprid into 6−CNA via guanidine and 5−hydroxy imidacloprid intermediates. Hence, *Bacillus cereus* presents a potential novel tool for removing the pesticide from contaminated water and soil. Isolation of the enzyme responsible for imidacloprid degradation can offer a viable method for commercial exploitation of purified enzymes. For successful commercial bioremediation setup, combined ecological knowledge, biochemical pathway and field engineering plans are necessary elements.

## Figures and Tables

**Table 1:** Experimental variables and the three levels of each factor used in the Box–Behnken design.

Independent Variables	Levels		
	Low (−1)	Centre (0)	High (+1)
A: pH	5	7	9
B: Imidacloprid Concentration (mM)	0.01	0.03	0.05
C: Agitation time (days)	1	12	21

**Table 2:** Decoded experimental Box–Behnken design used to evaluate experimental variables for biodegradation of imidacloprid by *Bacillus cereus*.

Run	Experimental Variables			Biodegradation (%)
	Factor A; pH	Factor B; Imidacloprid Conc. (mM)	Factor C; Agitation Time (Days)	
1	9	0.05	11	55
2	7	0.05	1	25
3	7	0.01	1	48
4	7	0.05	21	87
5	9	0.03	21	80
6	5	0.03	21	69
7	7	0.03	11	92
8	7	0.01	21	86
9	5	0.03	1	12
10	5	0.05	11	41
11	9	0.03	1	22
12	5	0.01	11	55
13	9	0.01	11	79

**Table 3:** Regression coefficients, R^2^ and probability values for imidacloprid biodegradation (* Significant at *p* < 0.05).

Regression Coefficient	Coefficient in Term of Variable Factors
Intercept	92.00
A-pH	−7.50 *
B-Imidacloprid conc.	26.88 *
C-Agitation time (days)	−2.50 *
AB	0.2500
AC	6.00
BC	−25.12
A2	−9.37 *
B2	−21.13
C2	−7.50 *
Mean	57.77
R^2^	0.9890
Adjusted R^2^	0.9559
Model F-value	29.92

## Data Availability

Data will be made available on request.
